# ASSESSING AT-RISK OLDER ADULTS THROUGH THE RAPID GERIATRIC ASSESSMENT

**DOI:** 10.1093/geroni/igz038.664

**Published:** 2019-11-08

**Authors:** Marla Berg-Weger, John Morley

**Affiliations:** Saint Louis University, St. Louis, Missouri, United States

## Abstract

With the need to increase gerontological competency among all health/social service professions, the Rapid Geriatric Assessment (RGA) (Morley, 2017) is a tool that can be used in most healthcare settings. Designed as a rapid screening, the validated RGA assesses frailty, sarcopenia, anorexia, cognitive function, and advance care planning. Developed in 2015 through the Geriatric Workforce Enhancement Program (GWEP), 3,489 students and 5,643 faculty and practitioners have been trained in its use and have completed 10,881 RGAs in case-finding, screening, nursing home, PACE, and hospital and residential settings. Non-population-based findings show higher-then-national prevalence in all four health condition areas across settings: frailty (n=3,140; 30%), sarcopenia (n=4,458; 42.1%), weight loss risk (n=3,012; 28.4%), and cognitive impairment (n=2,509; 23.7%). Only 34.8% of the total sample had completed advance care plans. Data comparing results by gender, age, race/ethnicity, and setting will be presented along with strategies for curricular and practice integration.

